# Desigual impacto de la COVID-19 en la mortalidad en residencias de personas mayores entre áreas de salud de Castilla-La Mancha

**DOI:** 10.23938/ASSN.1160

**Published:** 2026-04-22

**Authors:** María Victoria Zunzunegui Pastor, Iñaki Rubio Mengual, Álvaro Villar Baile, Fernando J. García López

**Affiliations:** 1 Escuela de Salud Pública Universidad de Montreal Quebec Canadá; 2 Universidad del País Vasco/Euskal Herriko Unibertsitatea España; 3 Centro Nacional de Epidemiología Instituto de Salud Carlos III Madrid España; 4 Centro de Investigación Biomédica en Red Enfermedades Neurodegenerativas (CIBERNED) España

**Keywords:** Mortalidad, COVID-19, Equidad en salud, Cuidados de larga duración, Castilla-La Mancha, Mortality, COVID-19, Health equity, Long-term care, Castilla-La Mancha

## Abstract

**Fundamento:**

Determinar si hubo desigualdades en la mortalidad por todas las causas y por COVID-19 en las residencias de personas mayores entre las áreas de salud de Castilla-La Mancha durante 2020, y si estas desigualdades se explican por la incidencia de COVID-19 en el municipio correspondiente.

**Métodos:**

Los datos de las residencias proceden del Portal de Transparencia de Castilla-La Mancha. Las residencias se clasificaron según área de salud, tamaño, gestión e incidencia de COVID-19 en el municipio. Utilizando regresión de Poisson, se estimaron las razones de riesgo de mortalidad por todas las causas y por COVID-19 según área de salud, considerando Toledo como referencia, ajustando por tamaño, gestión e incidencia municipal de COVID-19.

**Resultados:**

Las 310 residencias (25.150 plazas) informaron 4.460 defunciones (2.821 por COVID-19). La mortalidad por todas las causas varió entre el 7% en las residencias de Talavera y el 26% en las de Ciudad Real. La mortalidad por COVID-19 varió entre el 4% en las de Puertollano y el 17% en las de Ciudad Real. En el modelo ajustado, las residencias en Ciudad Real, Guadalajara, Albacete y Puertollano tuvieron mayor riesgo de mortalidad por todas las causas que las de Toledo, y las de Talavera, un riesgo menor. Las residencias de Albacete tuvieron mayor riesgo de mortalidad por COVID-19 que las de Toledo.

**Conclusión:**

En 2020 se confirmaron las desigualdades territoriales en el riesgo de morir en las residencias de mayores de Castilla La Mancha, solo parcialmente explicadas por la incidencia local de COVID-19.

## Abstract (en)

**Background:**

To assess whether inequalities existed across the eight health areas of Castilla-La Mancha (Spain) in all-cause and COVID-19 mortality in long-term care homes during 2020, and whether these differences were explained by COVID-19 incidence in the corresponding health areas.

**Methods:**

Data were obtained from the Castilla-La Mancha Transparency Portal. Care homes were classified by health area, size, ownership, and COVID-19 incidence at the health-area level. Poisson regression models were used to estimate risk ratios for all-cause and COVID-19 mortality in each health area, using Toledo as the reference, and adjusting for size, ownership, and health-area COVID-19 incidence.

**Results:**

Among 310 care homes (25,150 places), 4,460 deaths were recorded, including 2,821 attributed to COVID-19. All-cause mortality ranged from 7% in care homes in Talavera to 26% in those in Ciudad Real, while COVID-19 mortality ranged from 4% in care homes in Puertollano to 17% in care homes in Ciudad Real. In adjusted analyses, care homes in Ciudad Real, Guadalajara, Albacete, and Puertollano had higher all-cause mortality risk than those in Toledo, whereas those in Talavera showed lower risk. Only care homes in Albacete showed higher risk of COVID-19 mortality compared with those in Toledo.

**Conclusions:**

Substantial territorial inequalities in mortality risk are observed in long-term care homes in Castilla-La Mancha for 2020. These inequalities were only partially explained by the community incidence of COVID-19.

## INTRODUCCIÓN

La pandemia de COVID-19 reveló enormes carencias en las instituciones sociosanitarias españolas^1^. Su impacto fue especialmente significativo en las residencias de personas mayores, a las que afectó de forma desproporcionada^2^. Entre los países de la OCDE, España destacó durante el primer periodo de la pandemia por la desprotección de las personas mayores y, especialmente, por la elevada mortalidad de aquellas que vivían en residencias^3^^-^^6^. Durante el año 2020 las residencias no recibieron prioridad ni para prevenir y controlar la COVID-19 ni para prestar la atención sanitaria necesaria a las personas que vivían o trabajaban en ellas^1^^-^^7^.

Los análisis de mortalidad por COVID-19 en España muestran heterogeneidad entre comunidades autónomas, tanto en población general^8^ como en la población en residencias. Según datos de los portales de transparencia de las diecisiete comunidades autónomas sobre la mortalidad por COVID-19 durante los meses de marzo y abril de 2020, el 8,3% de los residentes en residencias de Castilla-La Mancha falleció por COVID-19, siendo la segunda comunidad autónoma con mayor mortalidad tras la Comunidad de Madrid.

Castilla-La Mancha (C-LM), tercera comunidad autónoma más extensa de España, tiene dos millones de habitantes. Alcanzada la autonomía que resultó del proceso de descentralización del Estado, asumió la competencia de los servicios sociales^9^ y sanitarios^10^. Su mapa sanitario delimita la población de los más de 900 municipios en ocho áreas de salud^11^. La gestión sanitaria se realiza a través de catorce gerencias de atención integrada^12^.

A nivel local, la prevención y el control de los brotes epidémicos en las residencias de personas mayores es competencia de los servicios provinciales de epidemiología (que dependen de la Dirección General de Salud Pública), de la atención primaria de salud (competencia del centro de salud de la zona donde está localizada la residencia) y de la atención especializada (hospital de referencia); estos dos últimos dependientes de la gestión local que se realiza en las gerencias de atención integrada. Los servicios residenciales dependen de la Consejería de Bienestar Social con delegaciones provinciales^13^.

Dados los múltiples niveles de toma de decisión en materia de servicios sanitarios y sociales en respuesta a la pandemia, cabe preguntarse si existieron desigualdades en la mortalidad en las residencias de C-LM según las áreas de salud y si estas desigualdades van más allá de lo explicable por los factores de riesgo de mortalidad identificados en la literatura, incluyendo la incidencia de COVID-19 en el municipio donde se ubicaba la residencia y las características estructurales y organizacionales de las residencias, así como su tamaño y las modalidades de gestión^14^^-^^19^.

Esta investigación se propone contribuir a la escasa evidencia científica sobre el impacto de la COVID-19 en la mortalidad de las personas que vivían en las residencias de C-LM antes de que la vacunación estuviera disponible. El objetivo de este estudio es examinar las desigualdades entre áreas de salud en la mortalidad en las residencias de personas mayores en Castilla-La Mancha durante el año 2020, y si estas desigualdades se explican por la incidencia de COVID-19 en el municipio donde se ubica la residencia.

## MÉTODOS

La hipótesis de este trabajo es que hubo desigualdades entre las áreas de salud de C-LM en la mortalidad por COVID-19 y por todas las causas en las residencias de personas mayores (residencias).

Se trata de un estudio transversal donde las unidades de análisis son las residencias. La fuente de datos de cada residencia fue el Portal de Transparencia de C-LM para el periodo entre el 1 de marzo 2020 y el 12 de enero 2021. La base de datos contenía información sobre fallecimientos y características de las residencias: nombre, número de plazas, dirección, municipio, titularidad y entidad gestora.

Con fines descriptivos, la mortalidad se examinó con dos indicadores, el porcentaje de mortalidad por COVID-19 y por todas las causas, que se obtuvieron dividiendo el número de defunciones por el número de plazas autorizadas, ya que se carecía de información sobre la ocupación, presumiblemente superior al 90% en marzo de 2020. El análisis de mortalidad por todas las causas se basó en las residencias de C-LM que aportaron datos sobre defunciones, mientras que el análisis de mortalidad por COVID-19 se basó en las residencias con información sobre defunciones por COVID-19, ya fuera confirmada por pruebas de infección activa o presencia de síntomas compatibles con la infección por COVID-19.

La variable de exposición en este estudio fue el área de salud. La población que vive en las residencias de estas áreas geográficas está sujeta a tres órganos locales de decisión política que pudieron influir en la gestión de la pandemia: la gerencia de atención integrada, la delegación provincial de salud pública y la delegación provincial de servicios sociales.

Las variables potenciales de confusión fueron el tamaño de las residencias, el tipo de gestión y la incidencia acumulada de COVID-19 en 2020 en el municipio de localización de cada residencia.

Las características de las residencias incluidas en los análisis fueron el tamaño medido por el número de plazas y la gestión clasificada en cinco tipos: privada con fines de lucro, privada sin fines de lucro, municipal con gestión indirecta o mixta, titularidad pública de la Junta de Comunidades de Castilla-La Mancha (JCCLM) y gestión privada, y titularidad pública y gestión pública (ambas de la JCCLM).

La incidencia acumulada de COVID-19 en 2020 en el municipio de cada residencia se estimó por la incidencia desde el inicio de la pandemia hasta el 21 de junio de 2020 (primer periodo) y desde el 22 de junio hasta el 6 de diciembre de 2020 (segundo periodo). Según el Atlas COVID, las incidencias se expresaron por 100.000 habitantes^20^. Aunque las incidencias municipales estaban desagregadas por tres grupos de edad -menores de 18, entre 18 y 65 y mayores de 65 años-, se decidió utilizar el valor de la incidencia en el grupo de edad entre 18 y 65 años por constituir la población en edad laboral que incluye a los trabajadores de las residencias, principal fuente de infección para los residentes. De esta forma, se excluyó a los residentes de la incidencia local, ya que contribuyen en gran parte de municipios a la incidencia poblacional de COVID-19.

### Análisis estadístico

Se examinó la forma funcional de la asociación entre la incidencia acumulada de COVID-19 por 100.000 habitantes en el municipio y las defunciones por COVID-19 y por todas las causas (dividiendo el número de defunciones por el número de residencias en el área de salud) mediante regresiones de Poisson cuyas variables predictoras fueron las incidencias acumuladas de COVID-19 por 100.000 habitantes en el municipio para el grupo de 18 a 65 años en el primer y en el segundo periodo de la pandemia. Se construyeron dos modelos con las variables continuas de incidencia, uno con un término lineal y otro con un polinomio con dos términos, uno lineal y otro cuadrático, y se mantuvo este segundo modelo por mostrar mejor bondad de ajuste. Para facilitar la interpretación de los coeficientes en la regresión, se aplicó una transformación logarítmica (Material suplementario S1, tablas S1_A_1 y S1_B_1).

Se evaluó el posible efecto de confusión del tipo de gestión estimando las asociaciones entre el tipo de gestión y las defunciones por COVID-19 y por todas las causas mediante regresiones de Poisson simples (Material suplementario S1, tablas S1_A_2 y S1_B_2).

La asociación de las defunciones por COVID-19 y por todas las causas con el área de salud se estimó -primero en un modelo sin ajustes y, posteriormente, ajustando el efecto de las variables de confusión (incidencia de COVID-19 en el municipio, tamaño y tipo de gestión de la residencia)- mediante modelos de regresión de Poisson, donde las defunciones fueron la variable dependiente y el factor de compensación (*offset*) fue el logaritmo neperiano del número de plazas. Se estimó la razón de riesgos (RR) de mortalidad (y su intervalo de confianza del 95%) mediante la exponenciación del coeficiente del área sanitaria, tomando el área de salud de Toledo como referencia por ser el área con mayor número de residencias (Material suplementario S2, tablas S2_A y S2_B). Se utilizó el paquete de análisis estadístico SPSS.v23.

## RESULTADOS

**Figura 1:**
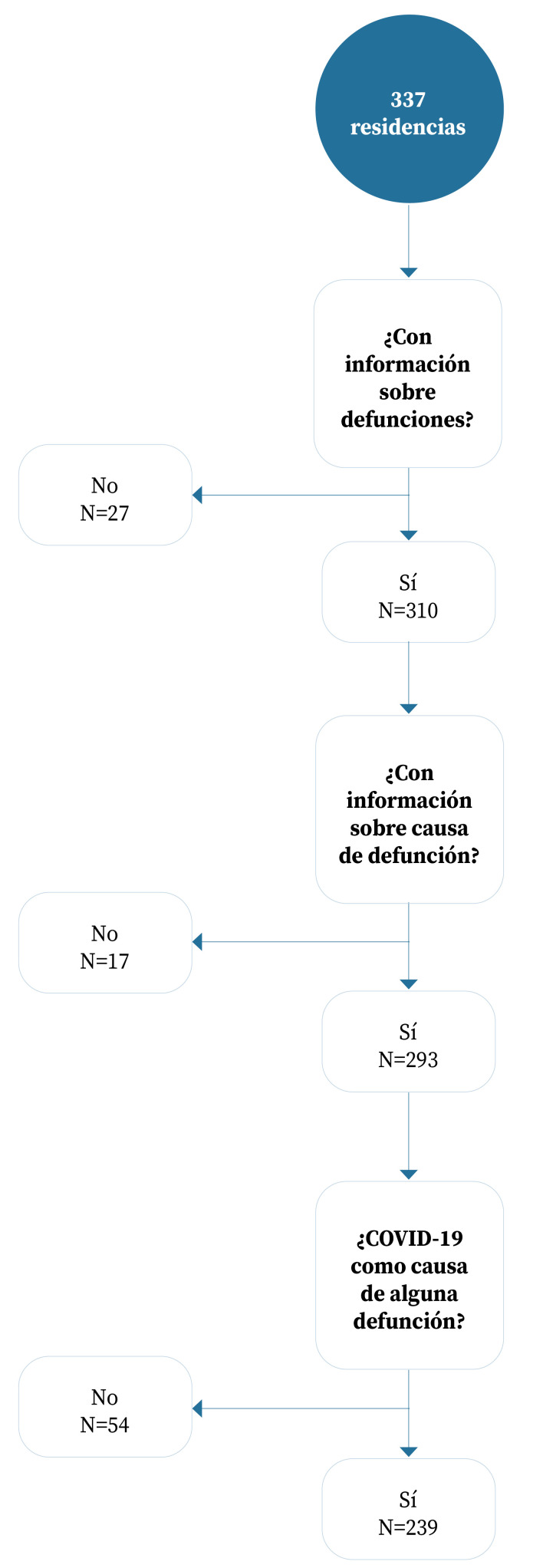
Diagrama de flujo de las 337 residencias de personas mayores de Castilla la Mancha según la disponibilidad de información sobre defunciones de residentes en 2020.

De las 337 residencias de C-LM, 27 no informaron datos sobre defunciones. De las 310 con datos sobre defunciones, 239 (77%) informaron haber registrado al menos una defunción por COVID-19, 54 informaron no haber tenido ninguna y 17 carecían de esta información. Por tanto, el análisis de mortalidad por COVID-19 se basó en las 239 residencias con información sobre defunciones por COVID-19 (Fig. 1).

El 39% de las 310 residencias de C-LM analizadas tenían gestión privada mercantil, el 23% eran privadas de entidades sin ánimo de lucro, el 18% eran municipales de gestión indirecta o mixta, el 14% eran públicas con titularidad de la JCCM y gestión indirecta, el 6% restante eran públicas de gestión pública.

En total había 25.150 plazas, con una media de 81 plazas por residencia, que no variaba significativamente entre áreas de salud. El 66% de las plazas estaban en entidades privadas (44% en mercantiles y 22% en entidades sin ánimo de lucro), el 29% en entidades municipales de gestión mixta, el 15% en residencias de las JCCM de gestión indirecta, y solo el 10% eran de las JCCM y/o de gestión pública.

La gestión se asoció al tamaño (p<0,001). El número medio de plazas era mayor en residencias de gestión pública que en las de gestión privada, mientras que las residencias municipales eran las que tenían menor número de plazas (Tabla 1).

**Tabla 1:** Tamaño y número medio de plazas de las residencias de mayores de Castilla-La Mancha en el año 2020, según su tipo de gestión

Área de salud	Residencias (N)	Plazas (Media)	Gestión				
			Privada		Mixta	Pública JCCM	
			Mercantil	Sin lucro	Municipal	Indirecta	Directa
Albacete	43	85	12	12	4	14	1
La Mancha-Centro	31	89	7	13	4	7	0
Ciudad Real	44	75	17	12	10	2	3
Cuenca	32	83	12	10	2	5	3
Guadalajara	41	85	22	6	4	6	3
Talavera de la Reina	45	72	23	8	10	2	2
Puertollano	12	77		2	3	0	1
Toledo	62	82	21	8	20	6	7
Total (N)	310		120	71	57	42	20
Plazas (Media)	25.150	81	92	77	40	91	126

JCCLM: Junta de Comunidades de Castilla-La Mancha.

Se registraron 4.460 defunciones en las 310 residencias de C-LM con datos de defunciones, 2.821 (63,3%) por COVID-19. El 82% de las 293 residencias con información disponible sobre COVID-19 como causa de defunción registraron al menos una defunción por COVID-19. Durante el periodo 2 se observaron mayores medianas de incidencia acumulada de COVID-19 (3.349 vs 670; p=0,002), y desigualdades significativas entre áreas de salud (Test Kruskal-Wallis p<0,001).

La media de defunciones por COVID-19 y por todas las causas fue 9,6 y 14,4, respectivamente, por residencia. La media de mortalidad por COVID-19 fue del 10,8% y varió entre el 3,6% en el área de Puertollano y el 16,6% en Ciudad Real. La mortalidad por todas las causas osciló entre el 6,6% en el área de Talavera de la Reina y el 26,3% en Ciudad Real (Tabla 2).

De acuerdo a los coeficientes de las regresiones de Poisson para las defunciones por COVID-19 y por todas las causas, las residencias municipales -que tenían el menor número de plazas- tenían significativamente menor riesgo de morir. La incidencia de COVID-19 en el municipio durante el periodo 1y 2 permanecieron significativamente asociadas a las defunciones por COVID-19 y por todas las causas en los modelos ajustados (Material suplementario, tabla S2).

Las estimaciones de las razones de riesgos de mortalidad por COVID-19 y por todas las causas mediante regresión de Poisson, antes y después del ajuste por las incidencias de COVID-19 en el municipio en los periodos 1 y 2 y por el tipo de gestión, se muestran en la Tabla 3.

**Tabla 2:** Incidencia acumulada de COVID-19 y media de defunciones y mortalidad en las residencias de Castilla-La Mancha según área de salud en 2020

Área de salud	Incidencia acumulada / 100.000 habitantes (Mediana)		Defunciones (Media)*		Mortalidad (%)	
	Periodo 1 (-21/junio)	Periodo 2 (22/junio-6/diciembre)	COVID-19 (N=293)	Todas las causas (N=310)	COVID-19 (N=293)	Todas las causas (N=310)
Albacete	572	3.000	14,7	21,5	14,2	22,2
La Mancha-Centro	1.154	2.951	10,8	16,2	10,8	16,5
Ciudad Real	947	2.779	12,2	19,8	16,6	26,3
Cuenca	1.297	3.642	8,5	15,2	9,8	17,7
Guadalajara	579	3.820	10,8	19,8	10,9	21,8
Talavera de la Reina	568	5.903	5	4,6	7	6,6
Puertollano	530	1.747	4,4	13,6	3,6	14,4
Toledo	489	4.207	6,7	8	7,4	8,8
Total	670	3.349	9,6	14,4	10,8	16,4

*: media de defunciones por residencia.

Antes del ajuste, las residencias de Albacete, La Mancha-Centro, Ciudad Real, Cuenca y Guadalajara tenían un riesgo de morir por COVID-19 significativamente superior a las del área de Toledo, mientras que las residencias de Talavera de la Reina y Puertollano tenían un riesgo inferior. Después del ajuste, solo las residencias del área de Albacete mantienen un riesgo de morir por COVID-19 significativamente mayor que las de Toledo (RR=2, IC95%: 1,1 a 5,4).

Las residencias de Puertollano mostraron un riesgo de defunción por todas las causas superior a las residencias de Toledo. En el modelo sin ajustar, el riesgo de mortalidad por todas las causas en las residencias en seis áreas (Albacete, La Mancha-Centro, Ciudad Real y Guadalajara, Cuenca, Puertollano) era mayor que en las de Toledo, y solo las de Talavera tenían un riesgo inferior. En el modelo ajustado, las residencias en Albacete, Ciudad Real y Guadalajara mantuvieron un riesgo de mortalidad por todas las causas significativamente mayor que las de Toledo, y las de Talavera riesgo inferior.

**Tabla 3:** Razones de riesgos de mortalidad por COVID-19 y por todas las causas estimadas mediante regresión de Poisson

	Mortalidad			
	COVID-19		Todas las causas	
Área sanitaria	RR (IC95%)	RRa (IC95%)	RR (IC95%)	RRa (IC95%)
Albacete	2,2 (2,1 a 2,3)	2,4 (1,1 a 5,4)	2,7 (2,5 a 2,8)	2,4 (1,5 a 3,8)
La Mancha-Centro	1,6 (1,5 a 1,7)	1,2 (0,5 a 2,8)	1,9 (1,8 a 2,0)	1,3 (0,8 a 2,3)
Ciudad Real	1,8 (1,7 a 1,9)	1,6 (0,7 a 3,5)	2,4 (2,3 a 2,5)	1,8 (1,2 a 2,9)
Cuenca	1,2 (1,1 a 1,3)	0,8 (0,3 a 1,8)	1,7 (1,6 a 1,8)	1,0 (0,6 a 1,7)
Guadalajara	1,6 (1,5 a 1,7)	1,7 (0,7 a 3,7)	2,3 (2,2 a 2,5)	2,0 (1,3 a 3,1)
Talavera de la Reina	0,7 (0,6 a 0,8)	0,9 (0,4 a 2,3)	0,5 (0,5 a 0,6)	0,5 (0,3 a 0,9)
Puertollano	0,7 (0,6 a 0,8)	1,3 (0,5 a 3,1)	1,7 (1,5 a 1,8)	1,9 (1,2 a 3,1)
Toledo	1,0	1,0		

RR: razón de riesgos bruta; RRa: razón de riesgos ajustada por las incidencias de COVID-19 en el municipio en los periodos 1 y 2 y por el tipo de gestión; IC: intervalo de confianza.

## DISCUSIÓN

En la medida de nuestro conocimiento, este estudio es el primer análisis del impacto de mortalidad en las residencias de personas mayores de C-LM durante la época prevacunal de la pandemia COVID-19. En ellas, dieciséis de cada cien residentes murieron entre marzo y diciembre de 2020 y once de estas dieciséis defunciones fueron registradas como COVID-19. El porcentaje de mortalidad por todas las causas en estos centros osciló entre el 6,6% y el 26,3% en las ocho áreas de salud.

Las desigualdades de mortalidad por COVID-19 en las residencias entre las áreas de salud son significativas, pero se atenúan cuando se tiene en cuenta la incidencia de COVID-19 en el municipio. Esto sugiere que las residencias no fueron protegidas adecuadamente frente a la COVID-19, a pesar de que se conocía que el riesgo de morir aumentaba con la edad y que vivir en viviendas colectivas facilita la transmisión de las infecciones respiratorias. Queda por explicar el doble riesgo de morir por COVID-19 de las residencias de Albacete, incluso teniendo en cuenta la incidencia local de COVID-19 y el tipo de gestión.

Los resultados del análisis de la mortalidad por todas las causas son algo diferentes. Las defunciones por todas las causas incluyen las defunciones por COVID-19 -las que fueron diagnosticadas y las que nunca lo fueron- y las defunciones por otras causas, que pudieron ser superiores a lo observado en periodos anteriores^21^. Los resultados del modelo sin ajustes muestran que las residencias en todas las áreas, excepto Talavera, tienen mayor riesgo de mortalidad que las de Toledo. En el modelo ajustado, solo las residencias en las áreas de Ciudad Real, Albacete, Guadalajara y Puertollano mantienen mayor riesgo de mortalidad (y las de Talavera menor riesgo) que la de las residencias de Toledo.

De acuerdo con otros autores^17^^,^^18^, la mortalidad por COVID-19 y por todas las causas en las residencias aumentaba consistentemente con la incidencia de COVID-19 en el municipio entre marzo y junio de 2020. En nuestro estudio, esta asociación se prolongó durante el segundo periodo, lo que sugiere que la infección de COVID-19 en la comunidad continuó teniendo un impacto significativo en la mortalidad de las residencias, contrariamente a lo observado en otros lugares donde a partir de julio mejoró la prevención y el control de la infección en las residencias^22^.

Una vez constatadas las desigualdades territoriales de mortalidad en residencias, formularemos una hipótesis considerando que el área de salud delimita el territorio donde se implementaron las decisiones sobre los servicios de salud pública, la asistencia sanitaria y los servicios sociales que recibirían los mayores que vivían en las residencias. Nuestra hipótesis es que la fragmentación de la atención sanitaria a las residencias -ya existente antes de la pandemia- y la multiplicidad de órganos de decisión pudieron haber dado lugar a variaciones locales en la gestión de la pandemia en las residencias de mayores que explicarían las desigualdades territoriales en su mortalidad. En apoyo de nuestra hipótesis aportamos las siguientes pruebas:

1. La escasez de documentación oficial sobre la gestión sanitaria para hacer frente a la pandemia COVID-19 en las residencias de C-LM, más allá de las instrucciones generales publicadas por el Ministerio de Sanidad^23^^,^^24^. El Portal de Transparencia facilitó acceso al informe titulado “Impacto de la pandemia sobre las residencias de personas mayores”, elaborado por la Consejería de Bienestar Social (disponible a los interesados), donde se explica que el 30 de marzo de 2020 se designaron cinco coordinadores provinciales y un coordinador regional con el mandato de cuantificar las necesidades y coordinar los apoyos sanitarios a los centros, pero no hemos encontrado guías de actuación sanitaria frente a la COVID-19 en residencias de mayores.
2. La escasez de camas hospitalarias y la falta de procedimientos para una gestión centralizada de camas. La sentencia judicial sobre el brote de la residencia Elder (Tomelloso, La Mancha Centro), ocurrido a primeros de marzo de 2020 y que ocasionó hasta fines de mayo 76 fallecidos de los 160 residentes activos a primeros de marzo^25^, relata fallos en la investigación del brote y en la atención sanitaria. Es evidente que el hospital de Tomelloso, con solo 78 camas, no podía atender la infección masiva de los 160 resientes de Elder, donde -como se lee en la sentencia- falló la prevención y el control del virus. Podría haber enviado pacientes al hospital general del área de La Mancha-Centro (320 camas), pero que también tenía dificultades; Maestre-Muñiz y col señalan dificultades para el traslado de los pacientes graves a otros hospitales por la ausencia de procedimientos^26^.
3. Posibles limitaciones en el acceso de los residentes a los hospitales en las áreas sanitarias con baja disponibilidad de camas. Presentamos a continuación cálculos para estimar el porcentaje de personas fallecidas por COVID-19 que no fueron trasladadas al hospital antes de morir entre marzo y mayo de 2020. El Instituto Nacional de Estadística registró 2.697 personas fallecidas en hospitales de C-LM y 1.766 en residencias. Maestre-Muñiz y col publicaron que, de las 142 personas fallecidas en el hospital de Alcázar de San Juan entre marzo y mayo de 2020, 31 procedían de residencias (21,8%). Aplicando esta proporción a los datos del INE, si el 21,8% de las 2.697 personas fallecidas en hospitales de C-LM entre marzo y mayo de 2020 procedía de residencias, 588 personas fallecidas procedían de residencias. Entonces podemos estimar que el total de residentes fallecidos por COVID-19 fueron los 1.766 residentes fallecidos en residencias y los 588 residentes fallecidos en hospital (2.354). Si el 75% de los residentes fallecidos por COVID-19 en ese periodo murieron en las residencias, ¿por qué solo se trasladó al hospital al 25% de los residentes que finalmente fallecieron?
4. Un estudio sobre la mortalidad en personas usuarias del Sistema de Atención a la Dependencia (SAD) de C-LM, incluyendo atención domiciliaria y residencial, reveló desigualdades en la mortalidad de las áreas de salud durante la pandemia: Ciudad Real destacaba por su alta mortalidad, mientras que Toledo y Talavera destacaron por la baja mortalidad de estos usuarios^13^.
5. Sobre las desigualdades intrarregionales durante la pandemia en la mortalidad de las personas mayores que vivían en residencias, cabe destacar la comparación de resultados entre algunas regiones de Ontario^22^ y entre dos provincias canadienses (la Columbia Británica y Ontario)^27^. Ambos estudios apuntan a desigualdades en la prevención y el control de infecciones y en la rapidez de respuesta sanitaria a los residentes infectados. En Italia se encontraron desigualdades intrarregionales en las hospitalizaciones de los residentes entre Toscana y Apulia que sugirieron desigualdades en la atención sanitaria disponible en las residencias de ambas regiones o desigualdades de acceso a los hospitales durante la pandemia^28^. Otros autores han documentado restricciones de acceso a la atención hospitalaria de los residentes o a la población mayor -tanto por COVID-19 como por otros trastornos crónicos- durante los primeros meses de la pandemia^29^^-^^31^.

Este estudio tiene varias limitaciones. Los datos que nos facilitó el Portal de Transparencia están incompletos. Desde marzo a diciembre de 2020, según el Instituto Nacional de Estadística, en C-LM ocurrieron 4.937 defunciones (1.937 por COVID-19 confirmado o posible) en las residencias, excluyendo los residentes que fallecieron después del traslado al hospital. Por ello, la comparación de los datos del Portal de Transparencia, que incluyen las defunciones en las residencias y en el hospital (4.427 defunciones, 2.788 por COVID-19), sugieren una subestimación y una mala clasificación de la causa de defunción. Por ejemplo, la mortalidad por COVID-19 (3,4%) en Puertollano parece subestimada, ya que la mortalidad por todas las causas fue elevada (14,4%). También carecemos de información sobre la edad de los residentes, pero el primer censo del IMSERSO, realizado después de la pandemia, informa que había 367 residencias de mayores en C-LM, el 75% de los residentes tenía más de 80 años y el 43% tenía dependencia en grado III, características similares a las de otras comunidades autónomas, lo que sugiere que las características de los residentes en las áreas de C-LM serían similares^32^.

Una evaluación después de la acción^33^ de la gestión de la pandemia de COVID-19 en las residencias de C-LM permitiría extraer lecciones para evitar desigualdades frente a la muerte en futuras epidemias.

En conclusión, hubo desigualdades territoriales considerables en la mortalidad de personas mayores que vivían en residencias según el área de salud de Castilla-La Mancha, y solo una parte de la desigualdad se explica por la incidencia de COVID-19 en la comunidad. Las agencias nacionales, regionales y locales deberían considerar las circunstancias epidemiológicas y organizativas locales al evaluar los resultados de las políticas implementadas para abordar la COVID-19.

## Data Availability

Repositorio de datos del Instituto de Salud Carlos III. https://repisalud.isciii.es/home
